# Validity of the Spanish-Language Patient Health Questionnaires 2 and 9: A Systematic Review and Meta-Analysis

**DOI:** 10.1001/jamanetworkopen.2023.36529

**Published:** 2023-10-17

**Authors:** Ashley Martinez, Semhar M. Teklu, Peggy Tahir, Maria E. Garcia

**Affiliations:** 1 Division of Nephrology, Department of Medicine, University of California, San Francisco, San Francisco; 2 Department of Pathology, University of California, San Francisco, San Francisco; 3 UCSF Library, University of California, San Francisco, San Francisco; 4 Division of General Internal Medicine, Department of Medicine, University of California, San Francisco, San Francisco; 5 Multi-Ethnic Health Equity Research Center, Division of General Internal Medicine, Department of Medicine, University of California, San Francisco, San Francisco; 6 Department of Epidemiology and Biostatistics, University of California, San Francisco, San Francisco

## Abstract

**Importance:**

Reliable screening for major depressive disorder (MDD) relies on valid and accurate screening tools.

**Objective:**

To examine the validity, accuracy, and reliability of the Spanish-language Patient Health Questionnaires 2 and 9 (PHQ-2 and PHQ-9) to screen for MDD.

**Data Sources:**

PubMed, Web of Science, Embase, and PsycINFO from data initiation through February 27, 2023.

**Study Selection:**

English- and Spanish-language studies evaluating the validity of the Spanish-language PHQ-2 or PHQ-9 in screening adults for MDD compared with a standardized clinical interview (gold standard). Search terms included *PHQ-2*,* PHQ-9*, *depression*, and *Spanish*.

**Data Extraction and Synthesis:**

Two reviewers performed abstract and full-text reviews, data extraction, and quality assessment. Preferred Reporting Items for Systematic Reviews and Meta-Analyses (PRISMA) guidelines were followed. Random-effects meta-analyses of sensitivity, specificity, and area under the curve (AUC) were performed. Internal consistency was evaluated using Cronbach α and McDonald ψ.

**Main Outcomes and Measures:**

Test accuracy and internal consistency. The PHQ-2 is composed of the first 2 questions of the PHQ-9 (targeting core depression symptoms of depressed mood and anhedonia; a score of 3 or higher (score range, 0-6) is generally considered a positive depression screen. If a patient screens positive with the PHQ-2, a follow-up assessment with the PHQ-9 and a clinical diagnostic evaluation are recommended. Once depression is diagnosed, a PHQ-9 score of 10 or higher (score range, 0-27) is often considered an acceptable threshold for treating depression.

**Results:**

Ten cross-sectional studies involving 5164 Spanish-speaking adults (mean age range, 34.1-71.8 years) were included; most studies (n = 8) were in primary care settings. One study evaluated the PHQ-2, 7 evaluated the PHQ-9, and 2 evaluated both the PHQ-2 and PHQ-9. For the PHQ-2, optimal cutoff scores ranged from greater than or equal to 1 to greater than or equal to 2, with an overall pooled sensitivity of 0.89 (95% CI, 0.81-0.95), overall pooled specificity of 0.89 (95% CI, 0.81-0.95), and overall pooled AUC of 0.87 (95% CI, 0.83-0.90); Cronbach α was 0.71 to 0.75, and McDonald ψ was 0.71. For the PHQ-9, optimal cutoff scores ranged from greater than or equal to 5 to greater than or equal to 12, with an overall pooled sensitivity of 0.86 (95% CI, 0.82-0.90), overall pooled specificity of 0.80 (95% CI, 0.75-0.85), and overall pooled AUC of 0.88 (95% CI, 0.87-0.90); Cronbach α was 0.78 to 0.90, and McDonald ψ was 0.79 to 0.90. Four studies were considered to have low risk of bias; 6 studies had indeterminate risk of bias due to a lack of blinding information.

**Conclusions and Relevance:**

In this systematic review and meta-analysis, limited available evidence supported the use of the Spanish-language PHQ-2 and PHQ-9 in screening for MDD, but optimal cutoff scores varied greatly across studies, and few studies reported on blinding schemes. These results suggest that MDD should be considered in Spanish-speaking individuals with lower test scores. Given the widespread clinical use of the tools and the heterogeneity of existing evidence, further investigation is needed.

## Key Points

**Question:**

Are the Spanish-language Patient Health Questionnaires 2 and 9 (PHQ-2 and PHQ-9) valid, accurate, and reliable depression screening instruments?

**Findings:**

In this systematic review and meta-analysis of 10 cross-sectional studies including 5164 Spanish-speaking adults, while sensitivity and specificity were generally high, optimal cutoff scores for the PHQ-2 and PHQ-9 were variable across studies. For the PHQ-2 studies, all identified an optimal cutoff score of less than 3 (a score ≥3 is typically used in US clinical settings).

**Meaning:**

These findings suggest that despite widespread use, there was limited available evidence in support of the Spanish-language PHQ-2 and PHQ-9, and optimal cutoff scores varied greatly; depression should be considered in Spanish-speaking patients with lower scores.

## Teaser

This systematic review and meta-analysis examines the validity, accuracy, and reliability of the Spanish-language Patient Health Questionnaires 2 and 9 to screen for major depressive disorder among Spanish-speaking adults.

## Introduction

Major depressive disorder (MDD) affects approximately 280 million people worldwide^1^ and 21 million people in the US,^2^ resulting in substantial psychosocial distress and disability.^3,4,5^ Accurate detection and diagnosis is important to initiating treatment and preventing the morbidity and early mortality associated with depression.^6,7,8^ The US Preventive Services Task Force recommends routine screening for depression in all adult primary care patients using validated screening tools such as the Patient Health Questionnaires 2 and 9 (PHQ-2 and PHQ-9),^9,10^ which are the most commonly used, widely validated, and practical depression screening tools in primary care settings and specialty mental health care settings in the US. The English-language versions of these tools require adequate language proficiency.

The instruments have been translated into many languages and are used in other countries and among linguistically diverse patients in the US. Worldwide, there are nearly 493 million Spanish native speakers, and in the US, Spanish is the second most common language spoken after English, with approximately 42 million speakers.^11^ In the US, studies on the prevalence of depression among Latino individuals have ranged from 15% to 30%,^12,13,14^ which is equal if not higher than among the general US population. Furthermore, Latino individuals with limited English proficiency have been found to have increased levels of mental health symptoms and unmet mental health service needs.^13,15,16,17^ Yet Spanish-speaking patients are less likely to have their depressive symptoms recognized and treated during routine clinical care.^18,19,20^ To improve recognition and treatment initiation as well as access to adequate mental health services, accessible and accurate depression screening tools must be available for Spanish speakers in the US. To be useful in primary care settings, depression screening tools must be valid (capable of measuring depression), accurate (measurement should be close to a true or accepted value), and reliable (instruments should yield the same results over multiple trials).

Two systematic reviews^21,22^ have previously examined depression screening questionnaires in Spanish; however, these reviews included only 1 study specifically examining the PHQ-9. Instead, these reviews evaluated lengthier tools, such as the Center for Epidemiologic Studies Depression Scale 20 and the Postpartum Depression Screening Scale, which are not practical in routine clinical care for the general adult population. Reuland et al^21^ included 1 study that explored the feasibility of using the Spanish version of the PHQ-9 in Honduran mothers; however, this study did not distinguish between MDD and perinatal depression, making it less applicable in primary care settings. Limon et al^22^ included a total of 4 studies published between 2009 and 2015, none of which examined the PHQ-2 or PHQ-9. Since 2015, there have been several PHQ-2– and PHQ-9–specific studies published,^23,24,25,26,27,28,29,30,31,32^ which prompts the need for an updated systematic examination of the literature.

The objective of our systematic review and meta-analysis was to evaluate the validity, accuracy, and reliability of the Spanish-language PHQ-2 and PHQ-9 as instruments for MDD screening, comparing these tools with standardized clinical interviews to inform adult depression screening in the US.

## Methods

### Patient Health Questionnaires (PHQ-2 and PHQ-9)

The PHQ-2 and the follow-up PHQ-9 consist of 2 and 9 items, respectively, assessing diagnostic symptoms of depression defined by the *The Diagnostic and Statistical Manual of Mental Disorders, Fifth Edition, Text Revision *(*DSM-5-TR*).^33^ The PHQ-2 is composed of the first 2 questions of the PHQ-9 (targeting core depression symptoms of depressed mood and anhedonia), and a score of 3 or higher (score range, 0-6) is generally considered a positive depression screen.^34^ If a patient screens positive with the PHQ-2, a follow-up assessment with the PHQ-9 and a clinical diagnostic evaluation are recommended. Once depression is diagnosed, a PHQ-9 score of 10 or higher (score range, 0-27) is often considered an acceptable threshold for treating depression.^34^

### Search Strategy

We searched PubMed, Web of Science, Embase, and PsycINFO databases to find relevant articles. The original search included records from database inception through December 20, 2020, and the search was updated on February 27, 2023. We used both index terms (MeSH and Emtree) and key words for our searches. We built the searches around these concepts: Patient Health Questionnaires (PHQ-2 and PHQ-9), screening for depression and depressive disorders, and the validity and accuracy of the questionnaires, focusing on the tools in Spanish. Multiple synonyms were developed for each concept to create broad searches, and there were no study date restrictions (the full search strategies for each database are available in eAppendix 1 in Supplement 1). Search terms included *PHQ-2*,* PHQ-9*, *depression*, and *Spanish*. We searched for studies published in the US and other Spanish-speaking regions. We also searched the references of articles selected for data extraction.

### Study Selection

Studies were included if (1) they included Spanish-speaking participants who were 18 years or older, (2) they evaluated the validity of the PHQ-2 or PHQ-9 in screening for MDD in Spanish, (3) the screening questionnaires were compared with standardized clinical interviews (considered the gold standard), (4) the publications reported peer-reviewed original research, and (5) they were conducted anywhere in the world but specified use of Spanish-language instruments. Gold standard clinical interviews included the Composite International Diagnostic Interview,^35^ the Mini International Neuropsychiatric Interview,^36^ the Schedules for Clinical Assessment in Neuropsychiatry,^37,38^ the Structured Clinical Interview for *DSM-III-R*,^39^ and the Primary Care Evaluation of Mental Disorders,^40^ which have all been validated for diagnosing depression.

We excluded studies evaluating the questionnaires as screening tools for disorders other than MDD (eg, perinatal depression), those without an acceptable reference standard for comparison, and those evaluating the scales in pediatric or non-Spanish–speaking populations. We excluded perspective pieces, editorials, and conference abstracts.

**Figure 1.**
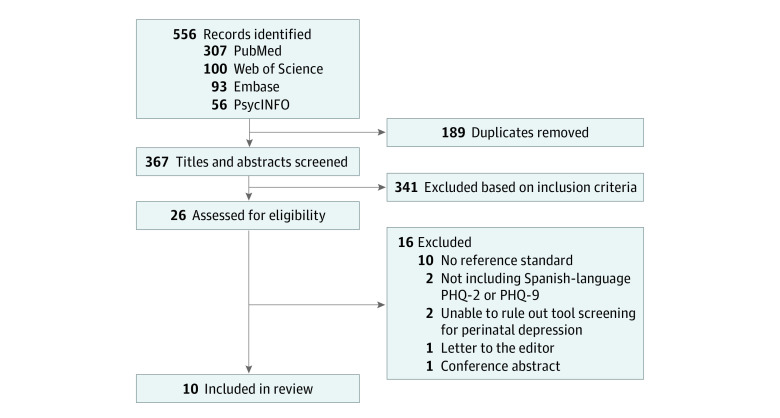
PRISMA Flow Diagram of Study Selection Reasons for study exclusion included validation of a depression screening tool was not the objective of the study (n = 288), the study was not focused on the Patient Health Questionnaire 2 (PHQ-2) or Patient Health Questionnaire 9 (PHQ-9) (n = 26), the study did not evaluate the Spanish language PHQ-2 or PHQ-9 (n = 9), the study focused on perinatal depression (n = 10), the study was conducted among patients younger than 18 years (n = 3), or the study was a duplicate record and removed manually during title and abstract screening (n = 5). PRISMA indicates Preferred Reporting Items for Systematic Reviews and Meta-Analyses.

Article titles and abstracts were independently reviewed by 2 reviewers (A.M. and S.M.T.) to determine whether studies fulfilled inclusion and exclusion criteria, with a third reviewer (M.E.G.) available to settle disagreements. Articles that met criteria were included for full-text review. If reviewers were unable to determine whether a study met inclusion criteria based on initial title and abstract review, the article was also included for full-manuscript review. This study followed the Preferred Reporting Items for Systematic Reviews and Meta-analyses (PRISMA) reporting guideline (Figure 1).^41^

### Data Extraction

Two reviewers (A.M. and S.M.T.) performed independent full-text reviews and data extraction using a standardized data extraction form. Data points extracted included publication year and author, years of study, country and clinical setting, screening tool evaluated (PHQ-2, PHQ-9, or both), gold standard clinical interview used for comparison, study design, index test and reference standard delivery and interpretation methods, participant demographics (ie, age and comorbidities), sample size, and psychometric properties and study outcomes, with a focus on measures of internal consistency (Cronbach α and McDonald ψ), index test optimal cutoff value, sensitivity, specificity, area under the curve (AUC), and prevalence of MDD via the gold standard interview. If necessary, we contacted study corresponding authors for clarification or to complete missing study information.

### Quality Assessment

Two reviewers (A.M. and S.M.T.) independently assessed the quality of the study design and methods of included studies, with a third reviewer (M.E.G.) available to settle disagreements. We used the revised Quality Assessment of Diagnostic Accuracy Studies (QUADAS-2)^42^; we tailored the tool for our study, as recommended by the tool developers.^42^ The QUADAS-2 is the most widely used guideline for the methodological assessment of systematic reviews and meta-analyses and is included in the *Cochrane Handbook for Systematic Reviews of Diagnostic Test Accuracy*.^43^ The assessment provides an organizational framework to evaluate the quality of heterogenous diagnostic accuracy studies. We conducted an assessment for risk of bias by evaluating 4 study domains: patient selection, index test, reference standard, and flow and timing. For our systematic review, we developed a ranking scale to grade each domain to produce a final ranking of each study’s overall risk of bias as low, high, or indeterminate. The full version of our tailored QUADAS-2 scale with detailed grading criteria is available in eAppendix 2 in Supplement 1.

### Statistical Analysis

A random-effects meta-analysis of proportions for the PHQ-2 and the PHQ-9, separately, was used to combine sensitivity and specificity for all studies. Analyses were subdivided by optimal cutoff scores (cutoff score of 1 or 2 for the PHQ-2 and ≥10 for the PHQ-9). Results were summarized in forests plots that include pooled effect size (sensitivity and specificity), 95% CIs, and weighted influence of each study on the overall meta-analysis. Separate random-effects meta-analyses for the PHQ-2 and the PHQ-9 were also performed for the AUC, also subdivided by questionnaire optimal cutoff scores. Meta-analyses for measures of internal consistency (Cronbach α and McDonald ψ) could not be conducted given that complete original study data were not available. In lieu of this, we detailed each study’s individual measures of internal consistency. We calculated Cohen weighted κ for interobserver agreement. All statistical analyses were performed using Stata/SE, version 17.0 (StataCorp LLC).

## Results

### Study Characteristics

Our search identified 367 distinct articles; 26 underwent full-text review, yielding 10 studies^23,24,25,26,27,28,29,30,31,32^ for this systematic review (Figure 1). One study^32^ focused solely on the PHQ-2, 7 studies^23,24,25,26,28,29,31^ evaluated the PHQ-9, and 2 studies^27,30^ examined both the PHQ-2 and PHQ-9. Two additional studies^44,45^ evaluating the PHQ-9 were excluded from this review given our inability to confirm that the tool was not used to screen for perinatal depression (Wulsin et al^44^ confirmed it was possible that some patients were in the perinatal period; Orive et al^45^ did not respond to personal communications). All studies^23,24,25,26,27,28,29,30,31,32^ included in this review used a cross-sectional study design. Most^23,24,25,26,27,29,30,31,32^ were published in English, with 1 study^28^ available only in Spanish; data were translated and abstracted by 1 author (A.M.), who is a bilingual and bicultural Spanish speaker. The characteristics and results for each study are shown in Table 1. After confirmatory emails were sent and responses reviewed, the Cohen weighted κ was 1 for the 2 reviewers (A.M. and S.M.T.).

**Table 1.** Characteristics and Results of Included Studies

Study	Mode of index test administration	Reference standard	Setting	Description of total study sample	Participants included in analysis, No.	Psychometric properties of index test
**PHQ-2 only**						
Scoppetta et al,^32^ 2021	Interview	MINI (MDE module)	Bucaramanga, Colombia; primary care centers; time frame not specified	243 Adults attending primary care centers; mean age: 34.1 y	243	Optimal cutoff value: ≥2; sensitivity: 0.87 (95% CI, 0.74-0.94); specificity: 0.74 (95% CI, 0.66-0.79); AUC: 0.89 (95% CI, 0.84-0.93); Cronbach α: 0.71 (95% CI, NR); McDonald ψ: 0.71 (95% CI, NR)
**PHQ-9 only**						
Aslan et al,^23^ 2020	Interview	CIDI (version 2.1)	Concepción and Talcahuano, Chile; 15 primary care health centers; July to August 2018	582 Chilean adults aged 65-80 y; mean age: 71.77 y	577	Optimal cutoff value: ≥6; sensitivity: 0.95 (95% CI, NR); specificity: 0.76 (95% CI, NR); AUC: 0.88 (95% CI, 0.85-0.90); Cronbach α: 0.78 (95% CI, 0.75-0.81); McDonald ψ: 0.79 (95% CI, 0.75-0.80)
Cassiani-Miranda et al,^24^ 2021	Interview	MINI	Bucaramanga, Colombia; primary care centers; time frame not specified	289 Adult primary care patients aged 18-65 y; mean age: 34.05 y	146	Optimal cutoff value: ≥7; sensitivity: 0.90 (95% CI, 0.81-0.99); specificity: 0.82 (95% CI, 0.76-0.87); AUC: 0.92 (95% CI, 0.88-0.96); Cronbach α: 0.80 (95% CI, NR); McDonald ψ: 0.81 (95% CI, NR)
Daray et al,^25^ 2019	Not specified	MINI (Spanish version 5.0)	Buenos Aires, Argentina; 3 hospitals; August 2013 to May 2014	306 Hospitalized adults aged ≥18 y; mean age: 54.15 y	257	Optimal cutoff value: 10; sensitivity: 0.81 (95% CI, NR); specificity: 0.79 (95% CI, NR); AUC: 0.87 (95% CI, 0.83-0.91); Cronbach α: 0.86 (95% CI, NR); McDonald ψ: NM
Limon et al,^26^ 2019^a^	Self	SCID	US; community health centers; time frame not specified	99 Latino adults with a farming or farming-adjacent occupation who lived in the US for <15 y; mean age: 38.44 y	99	Optimal cutoff value: 10; sensitivity: 0.99 (95% CI, NR); specificity: 0.67 (95% CI, NR); AUC: 0.93 (95% CI, NR); Cronbach α: 0.81 (95% CI, NR); McDonald ψ: NM
Muñoz-Navarro et al,^31^ 2017	Self	SCID	Valencia, Albacete, Vizcaya, and Mallorca, Spain; primary care centers; January to December 2014	260 Adults aged 18-65 y; mean age: NR	178	Optimal cutoff value: 12; sensitivity: 0.84 (95% CI, 0.77-0.90); specificity: 0.78 (95% CI, 0.64-0.87); AUC: 0.89 (95% CI, NR); Cronbach α: NM; McDonald ψ: 0.89
Saldivia et al,^28^ 2019	Self	CIDI (version 2.1)	Concepción, Chile; primary care centers; time frame not specified	1738 Adults aged 18-75 y; mean age: 54.52 y	1738	Optimal cutoff value: 7; sensitivity: 0.80 (95% CI, NR); specificity: 0.77 (95% CI, NR); AUC: 0.86 (95% CI, 0.83-0.88); Cronbach α: 0.89; McDonald ψ: 0.90 (95% CI, NR)
Urtasun et al,^29^ 2019	Self	MINI (Spanish version 5.0)	Buenos Aires, Argentina; primary care clinics and specialty mental health outpatient facilities; December 2013 to March 2014	169 Adult ambulatory care patients aged ≥21 y with and without depression; mean age: 47.4 y	169	Optimal cutoff value: ≥8; sensitivity: 0.88 (95% CI, NR); specificity: 0.87 (95% CI, NR); AUC: 0.87 (95% CI, 0.82-0.92); Cronbach α: 0.87 (95% CI, NR); McDonald ψ: NM
**PHQ-2 and PHQ-9**						
Errazuriz et al,^30^ 2022	Face to face but otherwise not specified	CIDI (CAPI version 3.0)	Santiago, Chile; household survey; August to October 2019	897 Spanish-speaking adult immigrants from Latin American countries aged ≥18 y; mean age: 36.6 y	PHQ-2: 897; PHQ-9: 897	PHQ-2: Optimal cutoff value: 1; sensitivity: 0.73 (95% CI, NR); specificity: 0.89 (95% CI, NR); AUC: 0.85 (95% CI, 0.67-1.00) Cronbach α: 0.75 (95% CI, NR); McDonald ψ: NM
						PHQ-9: Optimal cutoff value: 5; sensitivity: 0.85 (95% CI, NR); specificity: 0.90 (95% CI, NR); AUC: 0.91 (95% CI, 0.83-1.00); Cronbach α: 0.90 (95% CI, NR); McDonald ψ: NM
Gómez-Gómez et al,^27^ 2022	Interview	CIDI (module E)	Andalusia, Aragon, the Balearic Islands, Castile and Leon, Catalonia, and Galicia, Spain; primary care centers; February 2017 to January 2018	860 Adult primary care users aged 45-75 y; mean age for PHQ-2: 57.66 y; mean age for PHQ-9: 58.35 y	PHQ-2: 859; PHQ-9: 860	PHQ-2: Optimal cutoff value: ≥2; sensitivity: 0.88 (95% CI, NR); specificity: 0.70 (95% CI, NR); AUC: 0.85 (95% CI, 0.82-0.89); Cronbach α: NM; McDonald ψ: NM
						PHQ-9: Optimal cutoff value: 8; sensitivity: 0.86 (95% CI, NR); specificity: 0.82 (95% CI, NR); AUC: 0.91 (95% CI, 0.87-0.95); Cronbach α: NM; McDonald ψ: 0.84 (95% CI, 0.82-0.85)

Abbreviations: AUC, area under the curve; CAPI, computer-assisted personal interview; CIDI, Composite International Diagnostic Interview; MDE, major depressive episode; MINI, Mini International Neuropsychiatric Interview; NM, not measured; NR, not reported; PHQ-2, Patient Health Questionnaire 2; PHQ-9, Patient Health Questionnaire 9; SCID, Structured Clinical Interview for *DSM-III-R*.

^a^ Although not clearly stated within the manuscript, use of the Spanish-language version of the PHQ-9 was confirmed via contact with the authors of the study.

The studies were conducted in a range of settings. One study^26^ was completed in the US, 3^23,28,30^ in Chile, 2^24,32^ in Colombia, 2^25,29^ in Argentina, and 2^27,31^ in Spain. Most studies (n = 8)^23,24,26,27,28,29,31,32^ were conducted in primary care centers; 1 study^25^ was conducted in hospital settings, and another^29^ recruited from both primary care clinics and a mental health outpatient facility. One study^30^ recruited a representative community sample of adults. Among 5164 Spanish-speaking adults across the 10 studies,^23,24,25,26,27,28,29,30,31,32^ the mean ages ranged from 34.1 to 71.8 years. Patient characteristics varied and included immigrant populations, farm workers, ambulatory care patients, and hospitalized patients. Study analysis sample sizes were variable (range, 99-1738; median [SD], 250 [521.5]; 1999 for PHQ-2 and 4921 for PHQ-9 studies).

There was high variability in Spanish-language versions of the instruments used. Scoppetta et al^32^ translated the PHQ-2 for the purposes of their study. Five studies^23,27,28,30,31^ used a version of the PHQ-9 originally tested in general hospital Spanish inpatients.^46^ Cassiani-Miranda et al^24^ developed a new version of the Spanish-language PHQ-9; independent certified translators first translated the original English version and then adapted it based on patient and expert observations to develop a new Colombian version of the questionnaire. Daray et al^25^ used the Argentinian Spanish-language version of the PHQ-9, which had been previously used and studied by Urtasun et al.^29^ Limon et al^26^ did not specify which version of the PHQ-9 was used but confirmed by the author that a Spanish-language version of the scale was used (detailed version origin was not specified). Regarding the reference standards of choice, 4 studies^23,27,28,30^ used the Composite International Diagnostic Interview, 4 studies^24,25,29,32^ used the Mini International Neuropsychiatric Interview, and 2 studies^26,31^ used the Structured Clinical Interview for *DSM-III-R*.

### Psychometric Properties and Meta-Analysis of the PHQ-2

#### Performance When Using the Optimal Cutoff Score

For the 3 studies^27,30,32^ evaluating the PHQ-2, all identified optimal cutoff values via analysis of a receiver operating characteristic curve. Optimal cutoff scores ranged from greater than or equal to 1 or greater than to equal to 2, with AUCs ranging from 0.85 to 0.89. The overall pooled sensitivity for the Spanish-language PHQ-2 was 0.89 (95% CI, 0.81-0.95), the overall pooled specificity was 0.89 (95% CI, 0.81-0.95), and the overall pooled AUC was 0.87 (95% CI, 0.83-0.90). Forest plots for PHQ-2 sensitivity, specificity, and AUC are provided in eFigures 1 and 2 in Supplement 1.

#### Internal Consistency

The studies included in this review relied on a combination of the Cronbach α and McDonald ψ for measures of internal consistency. Values greater than 0.7 are generally considered to be acceptable for both the Cronbach α and the McDonald ψ, though there is substantial debate regarding the acceptability of these standard cutoffs for clinical rather than research purposes.^47,48^ For the Spanish-language PHQ-2, 2 studies^30,32^ reported Cronbach α (0.71^32^ and 0.75^30^), and 1 study^32^ measured the McDonald ψ, which was 0.71.

### Psychometric Properties and Meta-Analysis of the PHQ-9

#### Performance When Using the Optimal Cutoff Score

**Figure 2.**
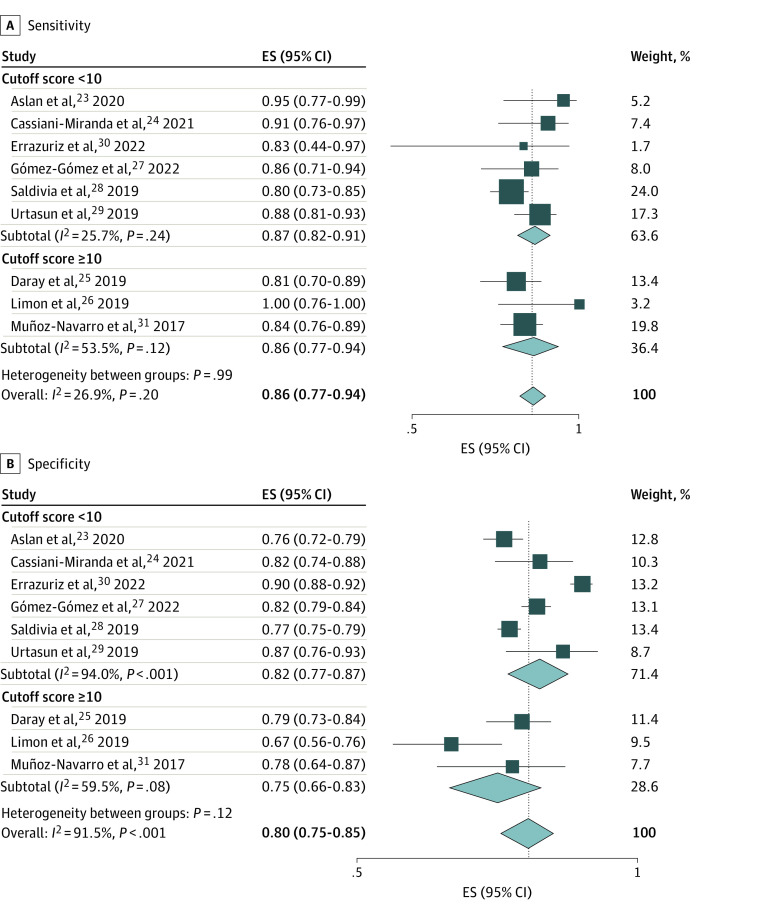
Meta-Analyses of the Sensitivity and Specificity of the Patient Health Questionnaire 9 by Cutoff Score Study estimates were obtained using a random-effects model. ES indicates effect size.

**Figure 3.**
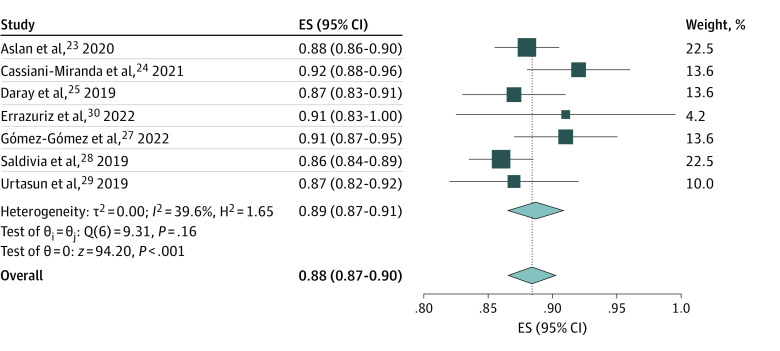
Meta-Analysis of the AUC of the Patient Health Questionnaire 9 Study estimates were obtained using a random-effects model. AUC indicates area under the curve; ES, effect size.

For studies evaluating the PHQ-9, 8 studies^23,24,25,27,28,29,30,31^ identified optimal cutoff values via receiver operating characteristic curve analysis. Optimal cutoff scores ranged from greater than or equal to 5 to greater than or equal to 12, with AUCs ranging from 0.86 to 0.92. One study^26^ used a cutoff score that was defined a priori. For the 6 studies^23,24,27,28,29,30^ that reported an optimal cutoff score of less than 10, our meta-analysis determined a pooled sensitivity of 0.87 (95% CI, 0.82-0.91), a pooled specificity of 0.82 (95% CI, 0.77-0.87), and a pooled AUC of 0.89 (0.87-0.91). For the 3 studies^25,26,31^ that reported an optimal cutoff score of 10 or higher, the pooled sensitivity was 0.86 (95% CI, 0.77-0.94), and the pooled specificity was 0.75 (95% CI, 0.66-0.83). Only 1 study^25^ with an optimal cutoff score of 10 or higher reported the 95% CI for AUC data that was necessary for meta-analysis (AUC, 0.87; 95% CI, 0.83-0.91). The overall pooled sensitivity was 0.86 (95% CI, 0.82-0.90), the overall pooled specificity was 0.80 (95% CI, 0.75-0.85), and the overall pooled AUC was 0.88 (95% CI, 0.87-0.90) for the Spanish-language PHQ-9 (Figure 2 and Figure 3).

#### Internal Consistency

Seven studies^23,24,25,26,28,29,30^ reported the Cronbach α, which ranged from 0.78 to 0.90. Four studies^23,24,28,31^ measured the McDonald ψ, which ranged from 0.79 to 0.90.

### Quality Assessment

While we identified no studies with high risk of bias in this review using the tailored QUADAS-2 tool, only 4 studies^24,25,31,32^ were graded as having low risk of bias. Six studies^23,26,27,28,29,30^ had an indeterminate risk of bias, largely owing to a lack of blinding information regarding the interpretation of the index test and reference standard. For example, 5 of the indeterminate studies^23,26,27,28,30^ did not elaborate as to whether the index test results were interpreted without the knowledge of the results of the reference standard or whether the reference standard was interpreted without the knowledge of the index test. For 6 studies,^23,24,25,27,31,32^ we were able to identify the interval between index test and reference standard administration (range, same day to within 2 weeks); however, the time frame was not delineated in the remainder of the studies.^26,28,29,30^ For 2 studies,^24,31^ only a subset of the study population received the reference standard and were included in the final analyses (due to study budget constraints or nondisclosed reasons). The quality and risk of bias evaluation for all studies is provided in Table 2.

**Table 2.** Quality Assessment of Included Studies

Question	Aslan et al,^23^ 2020	Cassiani-Miranda et al,^24^ 2021	Daray et al,^25^ 2019	Errazuriz et al,^30^ 2022	Gómez-Gómez et al,^27^ 2022	Limon et al,^26^ 2019^a^	Muñoz-Navarro et al,^31^ 2017	Saldivia et al,^28^ 2019	Scoppetta et al,^32^ 2021	Urtasun et al,^29^ 2019
Overall risk of bias	Indeterminate	Low	Low	Indeterminate	Indeterminate	Indeterminate	Low	Indeterminate	Low	Indeterminate
Was a consecutive or random sample of patients enrolled?	Yes: random	Yes: consecutive	Yes: consecutive	Yes: multistage random probability	Yes: random	Unclear	Yes: consecutive	Yes: random	Yes: consecutive	No: purposeful quota sampling^b^
Was a case-control design avoided?	Yes	Yes	Yes	Yes	Yes	Yes	Yes	Yes	Yes	Yes
Did the study avoid inappropriate exclusions?	Yes	Yes	Yes	Yes	Yes	Yes	Yes	Yes	Yes	Yes
Were the index test results interpreted without knowledge of the results of the reference standard (ie, blinded)?^c^^,^^d^	Unclear	Yes	Yes	Unclear	Unclear	Unclear	Yes	Unclear	Yes	Yes
Are the specificity and sensitivity recorded for multiple cutoff scores?	Yes	Yes	Yes	Yes	Yes	No	Yes	Yes	Yes	Yes
Was an appropriate version of the index test used?	Yes	Yes	Yes	Yes	Yes	Unclear	Yes	Yes	Yes	Yes
Is the reference standard likely to correctly classify the target condition?	Yes	Yes	Yes	Yes	Yes	Yes	Yes	Yes	Yes	Yes
Were the reference standard results interpreted without knowledge of the results of the index test (ie, blinded)?	Unclear	Yes	Yes	Unclear	Unclear	Unclear	Yes	Unclear	Yes	Yes
Was there an appropriate interval between index tests and reference standard?	Yes: same day	Yes: same day	Yes: same day	Unclear	Yes: same day	Unclear	Yes: <2 weeks	Unclear	Yes: same day	Unclear
Did all patients receive a reference standard?	Yes	No^e^	Yes	Yes	Yes	Yes	No^f^	Yes	Yes	Yes
Did patients receive the same reference standard?	Yes	Yes	Yes	Yes	Yes	Yes	Yes	Yes	Yes	Yes
Were all patients included in the analysis without a significant percentage of dropouts?	Yes	Yes	Yes	Yes	Yes	Yes	Yes	Yes	Yes	Yes

^a^ Although not clearly stated within the manuscript, use of the Spanish-language version of the Patient Health Questionnaire 9 (PHQ-9) was confirmed via contact with the authors of the study.

^b^ Purposeful quota sampling refers to a nonprobability sampling method by which investigators select participants based on particular characteristics.

^c^ Index test was the PHQ-2 or PHQ-9.

^d^ Reference standard was defined as the gold standard clinical interview used by the particular study.

^e^ Only 146 out of 243 patients received the reference standard.

^f^ Only 178 out of 260 patients received the reference standard.

## Discussion

In this systematic review and meta-analysis of the literature, we identified 10 studies^23,24,25,26,27,28,29,30,31,32^ examining the validity of the Spanish-language PHQ-2 and PHQ-9 in screening for MDD across diverse clinical settings. Limited available evidence supported the use of the Spanish-language PHQ-2 and PHQ-9 in screening for depression in Spanish-speaking patient populations; however, optimal cutoff scores varied greatly across studies, few studies reported on blinding schemes, and there was heterogeneity on PHQ-2 and PHQ-9 versions used. Given the large population of Spanish speakers in the US^11^ and the US Preventive Services Task Force recommendation^49^ for routine annual depression screening with tools such as the PHQ-2 and PHQ-9, these findings warrant further investigation. Additionally, our results could impact preferred depression screening tools for Spanish-speaking patients all over the world.

None of the studies that evaluated the PHQ-2^27,30,32^ found the optimal cutoff score to be 3, as is generally considered a positive screen for depression in the US.^34,49^ Instead, the Spanish-language PHQ-2 validation studies^27,30,32^ reported lower optimal cutoff scores, ranging from less than or equal to 1 to lower than or equal to 2; we could therefore be missing large numbers of Spanish-speaking patients with depression. Likewise, for the Spanish-language PHQ-9, optimal cutoff scores ranged from 5 to 12,^23,24,25,26,27,28,29,30^ with only 2 studies^25,26^ identifying 10 as the optimal cutoff score, such as was identified for the original English-language tool^10^ and repeat validation studies in primary care settings for English-speaking patients.^50^ The variability in PHQ-2 and PHQ-9 cutoff scores could reflect systemic differences between Spanish- and English-speaking populations, methodological differences in the studies evaluating the Spanish-language tool, or a combination of these factors.

The original studies that validated the English-language PHQ-2 and PHQ-9 by Kroenke et al^9,10^ were based on 6000 patients in 8 primary care and 7 obstetrics and gynecology clinics, of whom a subset of 580 primary care patients received a gold standard interview for criterion validity. An optimal cutoff score of 3 or higher for the PHQ-2 was found to have a sensitivity of 83% and a specificity of 92% for MDD (for the PHQ-9: optimal score ≥10, sensitivity of 88%, and specificity of 88%). A study by Arroll et al^50^ later validated the PHQ-2 and PHQ-9 in an even larger sample of 2642 primary care patients, finding a sensitivity of 86% and specificity of 78% for a PHQ-2 score of 2 or higher (sensitivity of 61% and of specificity 92% for PHQ-2 score ≥3) and a sensitivity of 74% and specificity of 91% for a PHQ-9 score of 10 or higher. The Spanish-language studies identified in this review were relatively small by comparison (N = 5164 for all studies^23,24,25,26,27,28,29,30,32^ in this review), which tempers conclusions that can be made with regard to screening test accuracy, reliability, and generalizability. For example, a systematic review of the Chinese-language PHQ-9 and PHQ-2,^51^ which concluded that the evidence was strong to support the validity of the Chinese-language scales, benefitted from larger sample sizes (N = 17 132; median [SD], 604 [742.5]).

Spanish-speaking individuals, who are substantially represented in the US population, are at high risk of undertreatment and underdiagnosis of depressive symptoms.^13,15,16,17,18,19,20^ Our systematic review found that the widespread use of the PHQ-2 and PHQ-9 in Spanish is based on limited data on the validity, accuracy, and reliability of these Spanish-language tools. Given the morbidity and early mortality associated with untreated depression,^3,4,5^ we need excellent tools to diagnose and monitor depressive symptoms among Spanish-speaking populations in the US and other countries.^52^ While the PHQ-2 and PHQ-9 will continue to be used widely, we need to understand the limitations of the available tools. As with the English-language tools, these measures do not replace the clinical diagnostic interview and patient-physician communication; depressive symptoms should be considered in Spanish-speaking individuals who score lower with these tools (particularly the PHQ-2, given that lower scores were uniformly used). Future research should include larger samples and study Spanish-language PHQ scales tailored to US Latino populations, recruiting in locations where they receive care (primary care clinics and community health centers) to avoid missed opportunities for diagnosis and intervention.

### Limitations

This review has limitations. Despite a thorough and systematic search of the literature, it is possible we missed some studies. We did not systematically search Spanish-language databases, for example, which may have revealed additional Spanish-language–focused studies. Furthermore, while our review was intended to inform depression screening for US Spanish-speaking patient populations, we found few studies of use of the Spanish-language tools in the US. Another limitation is the high rate of unknown blinding which resulted in indeterminate risk of bias for many of our included studies.^23,26,27,28,29,30^ This unknown information could affect the accuracy of results but reflects the current scope of knowledge about these widely used tools. Similarly, there was large heterogeneity in the versions of the Spanish-language PHQ scales used by the studies, making it difficult to compare the large number of Spanish-language versions likely being used in different clinical practices throughout the US. Use of different versions could further affect the construct validity of the PHQ-2 and PHQ-9, particularly among different Spanish-speaking cultural groups. Additionally, given the paucity of studies identified in this review, we were unable to evaluate the performance of the PHQ-2 and PHQ-9 compared with each standardized clinical interview, which may be understudied in Spanish-speaking or specific subpopulations.^21,53,54^ Finally, of all 10 studies^23,24,25,26,27,28,29,30,32^ in this review, not 1 measured the test-retest reliability; thus, conclusions on consistency of screening tests through time are difficult to make.

## Conclusions

This systematic review and meta-analysis found that limited available evidence supported the use of the Spanish-language PHQ-2 and PHQ-9 in screening for MDD, but optimal cutoff scores varied greatly across studies, and few studies reported on blinding schemes. Major depressive disorder should be considered in Spanish-speaking individuals with lower test scores. Given the widespread clinical use of the tools and the heterogeneity of existing evidence, further investigation is needed.

## Notes

Supplement 1. **eAppendix 1.** PHQ-9 PHQ-2 Questionnaire Spanish: Search Appendix **eAppendix 2.** Tailored QUADAS-2 Tool **eFigure 1.** Meta-Analyses of Sensitivity and Specificity of the PHQ-2 by Cutoff Score **eFigure 2.** Meta-Analysis of the AUC of the PHQ-2

Supplement 2.
Data Sharing Statement
