# Supplementary material for: Validity of the Spanish-Language Patient Health Questionnaires 2 and 9: A Systematic Review and Meta-Analysis
Source: JAMA Netw Open. 2023 Oct 17;6(10):e2336529. doi: 10.1001/jamanetworkopen.2023.36529 (PMC10582786; doi:10.1001/jamanetworkopen.2023.36529)
Supplement: Supplement 1. — eAppendix 1. PHQ-9 PHQ-2 Questionnaire Spanish: Search Appendix eAppendix 2. Tailored QUADAS-2 Tool eFigure 1. Meta-Analyses of Sensitivity and Specificity of the PHQ-2 by Cutoff Score eFigure 2. Meta-Analysis of the AUC of the PHQ-2 [file jamanetwopen-e2336529-s001.pdf]

Supplementary Online Content

Martinez A, Teklu SM, Tahir P, Garcia ME. Validity of the Spanish-language Patient Health Questionnaires 2 and 9: a systematic review and meta-analysis. *JAMA Netw Open*. 2023;6(10):e2336529. doi:10.1001/jamanetworkopen.2023.36529

eAppendix 1. PHQ-9 PHQ-2 Questionnaire Spanish: Search Appendix

eAppendix 2. Tailored QUADAS-2 Tool

eFigure 1. Meta-Analyses of Sensitivity and Specificity of the PHQ-2 by Cutoff Score

eFigure 2. Meta-Analysis of the AUC of the PHQ-2

This supplemental material has been provided by the authors to give readers additional information about their work.

© 2023 Martinez A et al. *JAMA Network Open*.

eAppendix 1. PHQ-9 PHQ-2 Questionnaire Spanish: Search Appendix

DATABASE	SEARCH STRATEGY
PubMed	("PHQ-9" OR "PHQ-2" OR “patient health questionnaire-9” OR “patient health questionnaire-2” OR "Patient Health Questionnaire"[Mesh]) AND ("Depression/diagnosis"[Mesh] OR depression screening OR depression assessment OR "Depressive Disorder/diagnosis"[Mesh] OR depressive disorder screening OR depressive disorder assessment) AND (efficacy OR reliability OR validity OR utility OR "Validation Studies as Topic"[Mesh]) AND (Spanish OR “Central America” OR “Central America”[Mesh] OR “Costa Rica” OR “Costa Rica”[Mesh] OR “El Salvador” OR “El Salvador”[Mesh] OR Guatemala OR “Guatemala”[Mesh] OR Honduras OR “Honduras”[Mesh] OR Nicaragua OR “Nicaragua”[Mesh] OR Panama OR “Panama”[Mesh] OR “Latin America” OR “Latin America”[Mesh] OR “South America” OR “South America”[Mesh] OR “Argentina”[mesh] OR Argentina OR “Bolivia”[Mesh] OR Bolivia OR “Chile”[Mesh] OR Chile OR “Colombia”[Mesh] OR Colombia OR “Ecuador”[Mesh] OR Ecuador OR “Guyana”[Mesh] OR Guyana OR “Paraguay”[Mesh] OR Paraguay OR “Peru”[Mesh] OR Peru OR “Uruguay”[Mesh] OR Uruguay OR “Venezuela”[Mesh] OR Venezuela OR “Mexico”[mesh] OR Mexico OR “Cuba”[mesh] OR Cuba OR “Puerto Rico”[mesh] OR “Puerto Rico” OR “Dominican Republic”[mesh] OR “Dominican Republic”)
Web of Science	("PHQ-9" OR "PHQ-2" OR “patient health questionnaire-9” OR “patient health questionnaire-2”) AND ("Depression diagnosis" OR depression screening OR depression assessment OR depressive disorder screening OR depressive disorder assessment) AND (efficacy OR reliability OR validity OR utility) AND (Spanish OR “Central America” OR “Costa Rica” OR “El Salvador” OR Guatemala OR Honduras OR Nicaragua OR Panama OR “Latin America” OR “South America” OR Argentina OR Bolivia OR Chile OR Colombia OR Ecuador OR Guyana OR Paraguay OR Peru OR Uruguay OR Venezuela OR Mexico OR Cuba OR “Puerto Rico” OR “Dominican Republic”)
Embase	('patient health questionnaire 2'/exp OR 'patient health questionnaire 2' OR 'patient health questionnaire 9'/exp OR 'patient health questionnaire 9') AND ('depression'/exp/dm_di OR 'depression assessment' OR 'depression screening') AND ('efficacy parameters'/exp OR 'efficacy parameters' OR 'efficacy'/exp OR efficacy OR 'validity'/exp OR validity OR 'reliability'/exp OR reliability OR utility) AND ('spanish'/exp OR Spanish OR 'central america'/exp OR 'central america' OR 'costa rica'/exp OR 'costa rica' OR 'el salvador'/exp OR 'el salvador' OR 'guatemala'/exp OR guatemala OR 'honduras'/exp OR honduras OR 'nicaragua'/exp OR nicaragua OR 'panama'/exp OR panama OR 'latin america'/exp OR 'latin america' OR 'south america'/exp OR 'south america' OR 'argentina'/exp OR argentina OR 'bolivia'/exp OR bolivia OR 'chile'/exp OR chile OR 'colombia'/exp OR colombia OR 'ecuador'/exp OR ecuador OR 'guyana'/exp OR guyana OR 'paraguay'/exp OR paraguay OR 'peru'/exp OR peru OR 'uruguay'/exp OR uruguay OR 'venezuela'/exp OR venezuela OR 'mexico'/exp OR mexico OR 'cuba'/exp OR cuba OR 'puerto rico'/exp OR 'puerto rico' OR 'dominican republic'/exp OR 'dominican republic')
PsycINFO	("PHQ-9" OR "PHQ-2" OR “patient health questionnaire-9” OR “patient health questionnaire-2”) AND ("Depression diagnosis" OR depression screening OR depression assessment OR depressive disorder screening OR depressive disorder assessment) AND (efficacy OR reliability OR validity OR utility) AND (Spanish OR “Central America” OR “Costa Rica” OR “El Salvador” OR Guatemala OR Honduras OR Nicaragua OR Panama OR “Latin America” OR “South America” OR Argentina OR Bolivia OR Chile OR Colombia OR Ecuador OR Guyana OR Paraguay OR Peru OR Uruguay OR Venezuela OR Mexico OR Cuba OR “Puerto Rico” OR “Dominican Republic”)

© 2023 Martinez A et al. *JAMA Network Open*.

eAppendix 2. Tailored QUADAS-2 Tool

Domain 1: Patient Selection
Could the selection of patients have introduced bias?
1A: Was a consecutive or random sample of patients enrolled?
1B: Was a case-control design avoided?
1C: Did the study avoid inappropriate exclusions?
Response options: yes/no/unclear

Domain 1 Risk for Bias Grading:
Low Risk: All domain 1 criteria are fulfilled.
High Risk: None of domain 1 criteria are fulfilled.
Indeterminate Risk: 1-2 domain 1 criteria are fulfilled.

Domain 2: Index Test
Could the conduct or interpretation of the index test have introduced bias?
2A: Were the index test results interpreted without knowledge of the results of the reference standard?
2B: Are the specificity and sensitivity recorded for multiple cutoff scores? (modified from QUADAS-2)
2C: Was an appropriate version of the index test used? (new from QUADAS-2)
Response options: yes/no/unclear

Domain 2 Risk for Bias Grading:
Low Risk: All domain 2 criteria are fulfilled.
High Risk: None of domain 2 criteria are fulfilled.
Indeterminate Risk: 1-2 domain 2 criteria are fulfilled.

Domain 3: Reference Standard
Could the reference standard, its conduct, or its interpretation have introduced bias?
3A: Is the reference standard likely to correctly classify the target condition?
3B: Were the reference standard results interpreted without knowledge of the results of the index test?
Response options: yes/no/unclear

Domain 3 Risk for Bias Grading:
Low Risk: All domain criteria are fulfilled.
High Risk: None of the domain criteria are fulfilled.
Indeterminate Risk: 1 domain 3 criteria is fulfilled.

Domain 4: Flow and Timing
Could the patient flow have introduced bias?
4A: Was there an appropriate interval between index tests and reference standard?
4B: Did all patients receive a reference standard?
4C: Did patients receive the same reference standard?
4D: Were all patients included in the analysis?
Response options: yes/no/unclear

Domain 4 Risk for Bias Grading:
Low Risk: 3-4 domain 4 criteria are fulfilled.
High Risk: None of domain 4 criteria are fulfilled.
Indeterminate Risk: 1-2 domain 4 criteria are fulfilled.

Overall Risk for Bias Determination
Grades from all individual domains for each study were compiled and then studies were ranked as follows in terms of overall risk for bias.
Overall low risk: All 4 domains were low risk.
Overall high risk: All 4 domains were high risk.
Overall indeterminate risk: Any grading that did not fall within the above compilations.

© 2023 Martinez A et al. *JAMA Network Open*.

eFigure 1. Meta-Analyses of Sensitivity and Specificity of the PHQ-2 by Cutoff Score

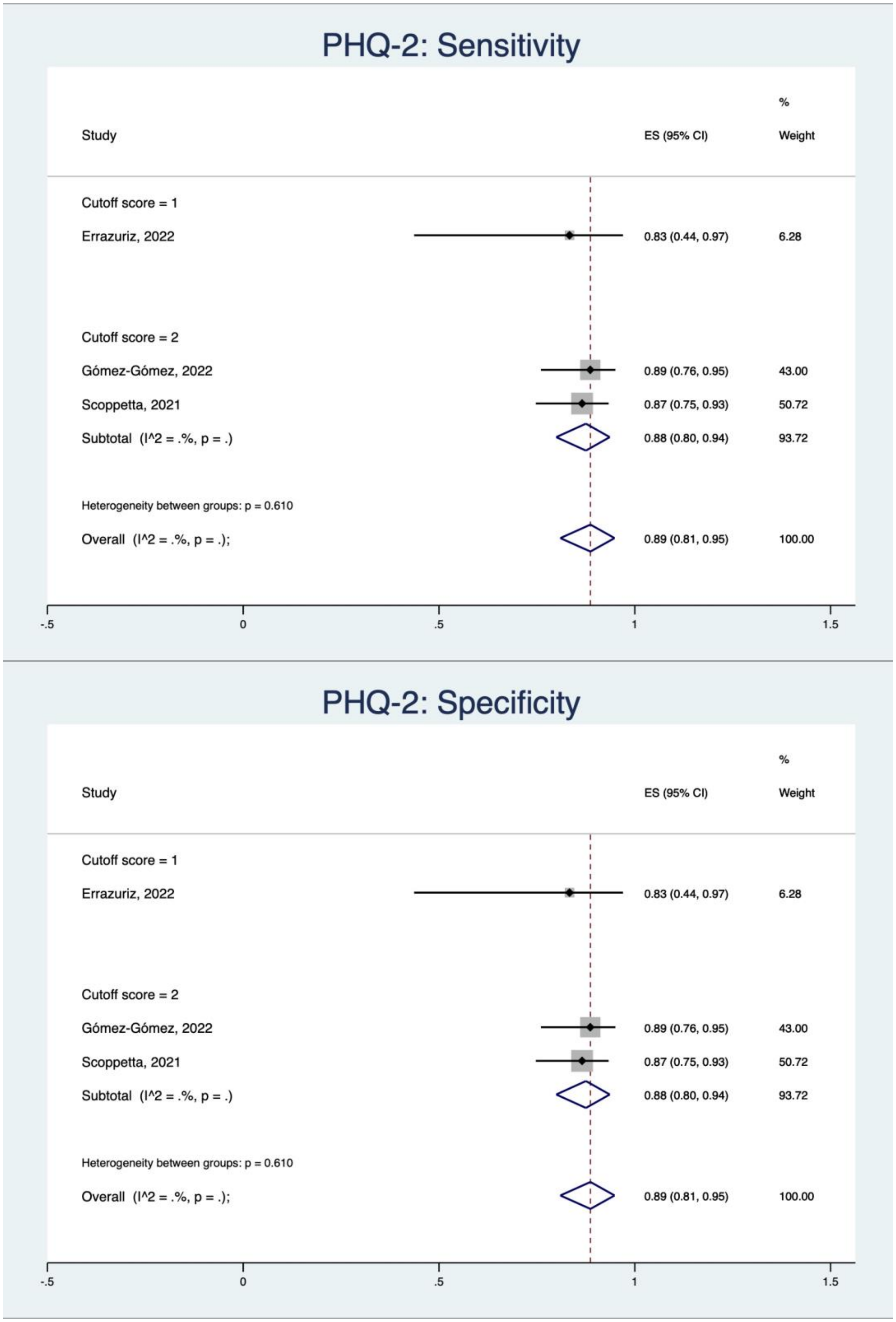

Studies are subdivided by optimal cutoff scores, either a score = 1 or 2 for the studies evaluating the PHQ-2. Study estimates were obtained using a random effects model. ES = effect size.

© 2023 Martinez A et al. *JAMA Network Open*.

eFigure 2. Meta-Analysis of the AUC of the PHQ-2

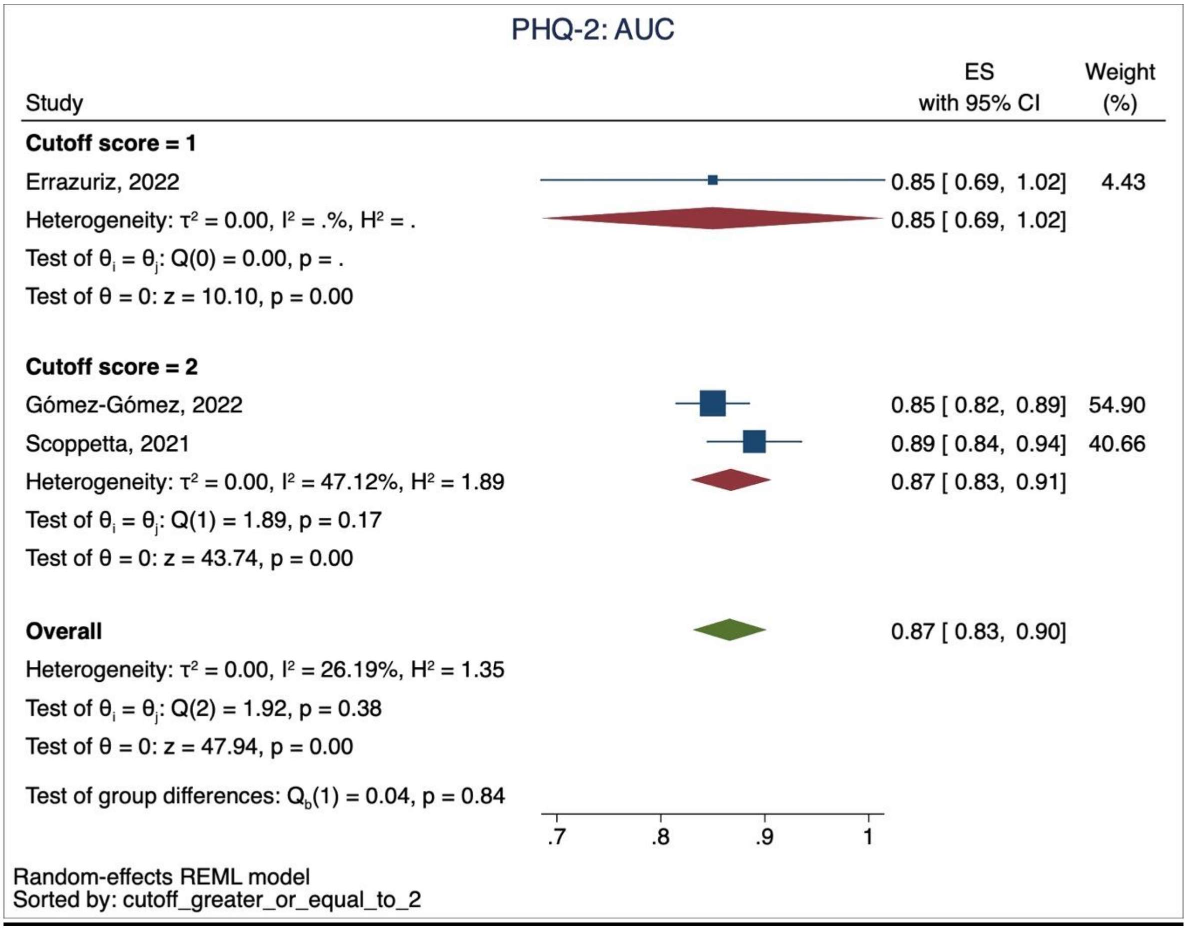

Studies are subdivided by optimal cutoff scores, either a score = 1 or 2 for the studies evaluating the PHQ-2. Study estimates were obtained using a random effects model. ES = effect size; AUC = area under the curve.

© 2023 Martinez A et al. *JAMA Network Open*.
