# Supplementary material for: Validity of the Spanish-Language Patient Health Questionnaires 2 and 9: A Systematic Review and Meta-Analysis
Source: JAMA Netw Open. 2023 Oct 17;6(10):e2336529. doi: 10.1001/jamanetworkopen.2023.36529 (PMC10582786; doi:10.1001/jamanetworkopen.2023.36529)
Supplement: Supplement 2. — Data Sharing Statement [file jamanetwopen-e2336529-s002.pdf]

Data Sharing Statement

Martinez. Validity of the Spanish-Language Patient Health Questionnaires 2 and 9. *JAMA Netw Open*. Published October 17, 2023. doi:10.1001/jamanetworkopen.2023.36529

Data
Data available: No

Additional Information
Explanation for why data not available: There was no primary data collection as part of this study.
